# *Campylobacter jejuni* Infection, Anti-Ganglioside Antibodies, and Neuropathy

**DOI:** 10.3390/microorganisms10112139

**Published:** 2022-10-28

**Authors:** Norman Latov

**Affiliations:** Department of Neurology, Weill Cornell Medicine, 1305 York Ave, Ste 217, New York, NY 10021, USA; nol2002@med.cornell.edu

**Keywords:** *Campylobacter jejuni*, lipopolysaccharide, Guillain Barre syndrome, peripheral neuropathy, neuropathy with IgM monoclonal gammopathy, anti-ganglioside antibodies, molecular mimicry

## Abstract

Preceding infection with *Campylobacter jejuni* (Cj) occurs in approximately 30% of patients with Guillain–Barre syndrome (GBS), and the risk of GBS following Cj infection is increased by 77 to 100-fold. GBS is most often of the axonal subtype and is thought to be mediated by IgG antibodies to peripheral nerve gangliosides that are cross reactive with oligosaccharides in the Cj lipopolysaccharides (LPS). The antibodies are thought to be induced by molecular mimicry, where immune reactivity to a cross reactive epitope in the infectious organism and normal tissue can cause autoimmune disease. Clonally restricted IgM antibodies that react with the same oligosaccharides in gangliosides and Cj-LPS are associated with chronic neuropathies of otherwise similar phenotypes. The anti-ganglioside antibodies in GBS are of the IgG1 and IgG3 subclasses, indicating T-cell reactivity to the same antigens that could help disrupt the blood–nerve barrier. Cj infection can activate multiple innate and adoptive pro-inflammatory pathways that can overcome immune tolerance and induce autoimmunity. Elucidation of the specific immune mechanisms involved in the development of the autoantibodies and neuropathy would help our understanding of the relation between infection and autoimmunity and aid in the development of more effective preventive interventions and therapies.

## 1. Introduction

Guillain Barre syndrome (GBS) is an acute onset, self-limiting, immune-mediated neuropathy that is often preceded by infection, most commonly *Campylobacter jejuni* (Cj) enteritis. The association was first reported by Rhodes and Tattersfield in 1982 [1], and additional reports soon followed [2]. In 1991, McKahnn and colleagues [3] reported on an acute paralytic disease of Children and young adults in northern China, referred to as “Chinese paralytic syndrome” that was subsequently identified as an axonal subtype of GBS [4,5]. Serological studies indicated a higher rate of preceding Cj infection than in controls [6]. In later studies, Ho and colleagues [7] reported that 66% of patients with GBS in Northern China had serological evidence of recent *C. jejuni* infection compared with 16% of village controls.

Antibodies that react with oligosaccharide epitopes of gangliosides are associated with both acute and chronic neuropathies. The association was first reported by Ilyas and colleagues in 1985 [8] in a patient with chronic neuropathy and monoclonal IgM that reacted with disialylated gangliosides, and subsequently in patients with GBS and IgG or IgM antibodies to LM1, GD1b, and GD1a/GT1b gangliosides [9]. Yuki and colleagues, in 1990 [10], reported the occurrence of IgG anti-GM1 antibodies in two patients with axonal GBS following Campylobacter enteritis, and Chiba and colleagues in 1992 [11] reported IgG anti-GQ1b antibodies in acute ataxic neuropathy with ophthalmoplegia or the Miller-Fisher syndrome (MFS) variant of GBS.

Moran and colleagues, in 1991 [12], and Aspinall and colleagues in 1992 [13] reported that Campylobacter jejuni (Cj) lipopolysaccharides (LPS) contain N-acetylneuraminic acid residues with the same linkages as in gangliosides. That was followed in 1993 by Yuki and colleagues’ report [14] that Cj-LPS contains GM1-like oligosaccharides, suggesting that the anti-GM1 antibodies in GBS following Cj infection may be induced by molecular mimicry or immune cross-reactivity between Cj-LPS and self-antigens.

The aim of this paper is to review current understanding of the relationship between CJ infection, anti-ganglioside antibodies, and neuropathy, the role of molecular mimicry, and the mechanisms responsible for the development of the autoimmune disease.

## 2. Epidemiologic Studies of Cj Infection in GBS

In a systematic review of published studies, approximately 30% of cases of GBS had evidence for preceding infection with *Campylobacter jejuni* [15], although with significant regional and seasonal variability of between 4.8 and 71.7 % [2]. The incidence of GBS following Cj infection was estimated as 30.4 to 117 per 100,000, or 77 to 100 times higher than the risk in the general population [16,17]. The neurological symptoms usually begin at 10 days to 3 weeks after the onset of diarrhea [2], whereas the mean excretion time of *C. jejuni* is 16 days [18], so that the presence of infection is usually established by serological studies, which remain positive for many weeks [19]. However, the serological studies can be negative in approximately 15% of culture positive subjects and remain elevated for many months, limiting the sensitivity and specificity of the studies [20].

## 3. Association of Preceding Cj Infection and Subtypes of GBS

GBS can present in its classical form as acute inflammatory demyelinating polyneuropathy (AIDP), or as one of its variant or subtypes. These include acute motor axonal neuropathy (AMAN), acute motor and sensory axonal neuropathy (AMSAN), acute ataxic neuropathy with ophthalmoplegia or Miller-Fisher syndrome (MFS), acute sensory or ataxic neuropathy, and overlap syndromes [21]. In studies examining the association of Cj with subtypes of GBS using electrophysiologic studies, Cj infection was most often [22,23,24,25,26,27,28,29], but not always [23], more frequently associated with the axonal than demyelinating subtypes.

The distinction between the axonal and demyelinating neuropathy is inexact; however, axons and myelin are closely intertwined in the peripheral nerves so that they are both frequently affected, with damage to one secondarily affecting the other. In electrodiagnostic studies, demyelination has been defined by the presence of conduction abnormalities that exceed those that can result secondarily to axonal loss, with the default diagnosis being axonal degeneration, so that intermediate cases, or those with demyelinating lesions in proximal segments that are inaccessible to evaluation, could be misclassified as axonal [30]. Demyelinated nerves can also become inexcitable [31], or undergo remyelination, with two to eight lamellae sufficient for recovery [32], in which case they could also be misclassified. As example, Bericano and colleagues [33,34] reported a case of apparent axonal GB, in whom pathological studies at postmortem revealed the presence of demyelinating changes proximally at the nerve roots, with secondary axonal degeneration in the more distal nerve segments. In some cases, the demyelinating changes are transient, so that the findings may depend on when in the study is conducted, in which case there is debate as to whether the neuropathy is primarily demyelinating or axonal [35,36]. The term nodo-paranodopathy has sometimes been used to describe ultrastructural alterations at the nodes of Ranvier that can involve both myelin and axons.

## 4. IgG Anti-Ganglioside Antibodies in GBS following Cj Infection

IgG anti-ganglioside antibodies were reported to be elevated in 41 to 85% of patients with GBS following Cj infection, most often, but not always with the axonal AMAN and ASMAN subtypes [7,22,25,26,28,29,37,38,39,40]. Anti-ganglioside antibodies can also be seen in GBS following other triggers or infections, but rarely with Cj infection in the absence of GBS [39,41]. The differences between studies could be due to environmental or host factors, genetic predisposition, other triggers of preceding infections, and differences in electrodiagnostic criteria or assay methodologies.

The IgG antibodies often cross-react with multiple gangliosides due to shared oligosaccharides, but several patterns or profiles can be recognized. The major profiles are: (1) anti-GM1 IgG antibodies to the Gal-GalNAc epitope that can cross react with GD1b, (2) anti-GD1a IgG antibodies to the NeuNAc-Gal epitope that can cross react with GM1, GT1b or GT1a, and (3) anti- GQ1b or disialylated gangliosides antibodies to the NeuNAc-NeuNAc-Gal epitope that can cross react with GD1b, GD3, and GT1a. The antibodies can be specific or cross react with one or more of the other gangliosides that share that epitope. IgG antibodies to GM1 or GD1a or their cross-reactive gangliosides are associated with the axonal subtypes or AMAN and AMSAN, whereas IgG anti-GQ1b or disialylated ganglioside antibodies are associated with MFS and bulbar or sensory variants [38,42]. The anti-ganglioside antibodies from patients with GBS often cross react with isolates of Cj from the same patients, providing additional evidence that the antibodies are induced by the infection via molecular mimicry [41].

## 5. IgM Anti-Ganglioside Antibodies cross React with C-LPS in Chronic Neuropathies

Monoclonal or polyclonal IgM antibodies that react with the same gangliosides as the IgG antibodies in GBS are associated with chronic neuropathy syndromes that otherwise resemble the acute GBS phenotypes [43,44]. The association of monoclonal or polyclonal IgM anti-GM1 antibodies with motor or multifocal motor neuropathy was first described by Freddo and colleagues in 1986 [45] and Pestronk and colleagues in 1988 [46], respectively, and the association of monoclonal IgM anti-GD1b and disialylated gangliosides with chronic sensory or ataxic neuropathy was first reported by Daune and colleagues in 1992 [47], with the full syndrome later being broadened to include ophthalmoplegia, and sometimes referred to as Chronic Ataxic Neuropathy with Ophthalmoplegia, IgM paraprotein, Cold Agglutinins, and anti-disialylated ganglioside antibodies (CANOMAD) [48]. The IgM anti-GM1 or GD1b antibodies in the chronic neuropathies also cross-react with Cj-LPS, similarly to the IgG antibodies in GBS [49,50,51].

## 6. Pathological Studies

Pathological studies of GBS at postmortem are consistent with antibody-mediated immunopathology. In AIDP, the earliest changes seen, before the invasion of macrophages or inflammatory cells, were that of vesicular degeneration and deposits of C3d and C5b-9 along the surface of Schwann cells [52]. Immunoglobulin deposits could not be ascertained due to background staining in the endoneurium. In AMAN, deposits of IgG and C3d were seen on the axolemma of large motor fibers, with macrophage infiltration [53], and in AMSAN, there was evidence for macrophage mediated axonal degeneration with few other inflammatory changes [5]. In Miller-Fisher syndrome (MFS), studies showed the presence of inflammatory demyelination, similar to classical GBS [54].

In chronic ataxic neuropathy with IgM anti-GD1b and disialylated ganglioside antibodies, pathological studies at post-mortem showed dorsal column and dorsal root atrophy with mixed mononuclear cell infiltration in one case [55], and sensory ganglionopathy, spinal dorsal columns atrophy, and infiltration of clonal B-lymphocytes in the cranial nerves, dorsal roots, and cauda equina in another [56]. In motor neuropathy with elevated IgM anti-GM1 antibodies, motor nerve biopsy in one patient revealed deposits of IgM and the complement activation product C3i on the motor nerve fibers, with focal macrophage infiltrates in the periaxonal space [57].

## 7. Role of Anti-Ganglioside Antibodies in the Neuropathy

In experimental animal studies, rabbits immunized with GM1 ganglioside or Cj-LPS bearing GM1-like oligosaccharides developed an acute axonal neuropathy with flaccid weakness and IgG anti-GM1 antibodies, resembling axonal GBS [58,59,60]. Rabbits immunized with GD1b developed acute sensory ataxic neuropathy with degeneration of the dorsal root ganglia and IgM anti-GD1b antibodies [61]. Passive transfer of monoclonal anti-ganglioside antibodies can cause acute axonal neuropathy in susceptible mice [62]. In humans, some patient developed GBS with anti-GM1 antibodies after parenteral administration of gangliosides [63].

In immunocytochemical studies, the distribution of gangliosides targeted by the antibodies generally correlates with the associated neuropathy. However, although peripheral nerve myelin and axons are rich in gangliosides, these are not always exposed at the surface or available for binding, so that results of immunocytochemical studies depend on tissue processing or fixation that can expose otherwise shielded epitopes [58,63,64,65]. Such shielded epitopes, however, might become exposed in the course of the neuropathy, contributing to the secondary demyelination or axonal degeneration.

In intraneural injection studies, where the structural integrity of the nerve is maintained, cholera toxin (CT), which is highly specific to GM1, bound to the nodes and paranodal myelin in the peripheral nerves [66]. The same pattern of staining was seen on frozen sections with IgG anti-GM1 antibodies from a patient with axonal GBS [63] and following intraneural injection of anti-GM1 IgM from a patient with multifocal motor neuropathy [67]. Targeting GM1 at the nodes of Ranvier by the intraneural injection of CT and anti-CT antibodies also caused a conduction block with axonal degeneration, as seen in AMAN or MMN [68]. Human IgM anti-GM1 antibodies were also shown to bind to the surface of isolated unfixed human motor neurons [65] and in tissue sections, human anti-GM1 IgM and IgG ntibodies bound to the pre-synaptic region at the terminus of the motor nerves [63,69,70].

In other immunocytochemical studies, several mouse monoclonal anti GD1a antibodies preferentially stained motor fibers, and an anti-GD1b antibody preferentially stained the large dorsal root ganglia [64]. A mouse monoclonal anti-GD1b antibody immunostained neurons in the dorsal root and sympathetic ganglia [71], an anti-GQ1b/GT1a antibody stained the paranodal regions of human oculomotor, trochlear and abducens nerves [72], and an anti-GQ1b/GT1a/GD1b antibody immunostained the motor endplates in human extraocular muscles and muscle spindles [73].

In physiologic studies, anti-GM1 antibodies were reported to block sodium channels [74,75,76] and to interfere with synaptic transmission [58,77,78,79]. Anti-GD1a antibodies altered presynaptic release and muscle action potentials [77], and anti-GT1a/GD2/GQ1b antibodies blocked synaptic transmission [80].

In other studies, a human IgM monoclonal anti-GD1b and disialylated gangliosides antibody induced cell death of cultured rat dorsal root ganglia [81], and human IgM monoclonal anti-GM1 antibodies caused degeneration of human spinal cord motor neurons in muscle co-cultures [82].

## 8. Role of the Blood–Nerve Barrier

Anti-ganglioside antibodies, by themselves, may not be sufficient to cause disease, as they can persist at high titer after the acute phase of the illness [83] and also occur in the absence of neuropathy [84]. Other factors, such as permeability of the blood–nerve barrier, pro or anti-complementary factors [85], macrophage activation, cytokines, or T-cell adhesion or reactivity [84], are likely to play a role in the development of neuropathy. In patients with GBS, the occurrence of T-cell-dependent IgG1 and IgG3 anti-ganglioside antibodies indicates the presence of reactive T-cells that could disrupt the blood–nerve barrier, contributing to the acute onset and severity of the disease.

**Figure 1:**
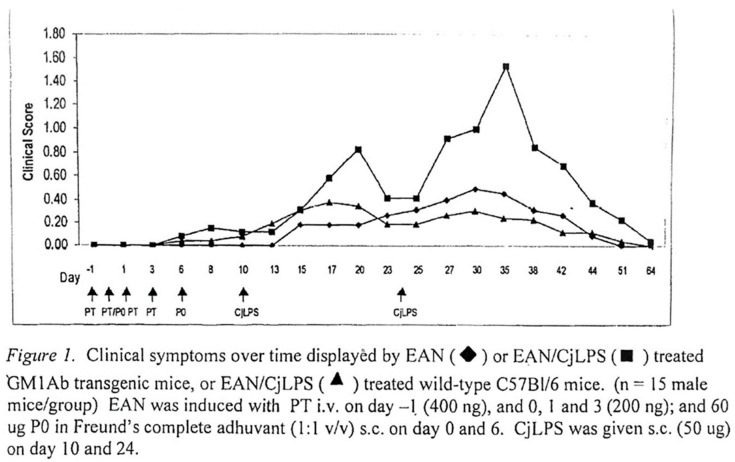
Experimental allergic neuritis (EAN) was induced in GM1 antibody transgenic mice (GM1Ab) by immunization with myelin PO peptide (180-199) in the presence of Freund’s adjuvant and pertussis toxin on days 0 and 6. On days 10 and 24 the mice received s.c. injections of LPS from C. jejuni 0:19 (CjLPS). Signs of neuropathy were scored from 0–6 (normal–death). Serum IgM anti-GM1 antibody were assayed by ELISA. Controls included similarly treated wild-type (WT) mice and GM1Ab mice given only EAN or CjLPS. EAN mice showed signs of peripheral neuropathy ranging from floppy tail (score 0.5–1), to moderate hind limb weakness (score 2.5). CjLPS induced IgM anti-GM1 antibody titers of >1:100,000 in EAN/GM1 Ab mice with earlier onset, greater severity and longer duration of clinical symptoms than in control groups. The mean day of onset was 13, 16 and 22 post primary PO injection. Duration of symptoms was 35, 22 and 18 days and clinical scores at day 35 (peak severity) were 1.54, 0.25, and 0.46 for the GM1Ab/EAN/LPS, control GM1Ab/EAN and WT/EAN/LPS groups respectively (*p* < 0.05). GM1 antibody titers in the GM1Ab/EAN/LPS and GM1Ab/LPS were >100-fold higher than in the GM1Ab/EAN group. No signs of neuropathy were seen in the GM1Ab/LPS group. High circulating levels of anti-GM1 antibodies caused a more severe neuropathy in EAN mice. (Lee, G., Willison, H.J., Latov, N. Anti-GM1 ganglioside antibodies increase severity of neuropathy following induction of experimental autoimmune neuritis. JPNS 2005, 10 (Suppl. 1), 51).

In experimental animal studies, the blood–nerve barrier can be disrupted by T-cells reactive with nerve antigens, allowing ingress of serum immunoglobulins [86]. Passive transfer of antibodies to galactocerebroside (GalC), a glycolipid in peripheral nerves, does not by itself cause neuropathy, but the addition of subclinical doses of T-cells reactive to myelin P2 protein to induce experimental allergic neuritis (EAN) resulted in overt clinical disease with markedly enhanced demyelination [87]. Of interest, although human IgM anti-GM1 antibodies do not react with GalC, some mouse or rabbit anti-GalC antibodies cross-react with GM1 [88] and patients with multifocal motor neuropathy have elevated titers of IgM anti-GM1/GalC complexes more frequently than to GM1 alone [89]. In transgenic mice expressing human IgM anti-GM1 antibodies, the induction of the anti-ganglioside antibodies by the administration of Cj-LPS does not by itself cause neuropathy, but the antibodies cause more severe disease when induced in EAN mice (Figure 1).

## 9. Immune Mechanisms and Induction of Anti-Ganglioside Antibodies

The IgM anti-GM1 antibodies in multifocal motor neuropathy can be monoclonal or polyclonal, with the polyclonal antibodies exhibiting restricted clonality [90,91]. The antibodies exhibit diverse V region gene usage with extensive somatic mutation, consistent with an antigen-driven response [92] The B-cells in IgM monoclonal gammopathies frequently exhibit mutations in the MYD88 gene that promote cell proliferation [93] and can be further stimulated by cross reactive antigens in the course of infection or autoimmune disease [94]. The chronic neuropathies begin insidiously, so it is not possible to know whether Cj is involved at the onset.

LPS is an immune stimulant that induces a proinflammatory response by activating the TLR4/ MYD88 pathway [95]. Cj-LPS can, in addition, engage the B-cell surface IgM anti-GM1 antigen receptor, providing an additional antigenic stimulus. As an example, transgenic B-cells expressing human IgM anti-GM1 antibodies are more responsive to stimulation by Cj-LPS bearing GM1-like oligosaccharide than by control LPS [96]. In another set of experiments, administration of Cj bearing the GalGalNAc epitope, which is the target of anti-GM1 antibodies, induced the production of anti-GalGalNAc antibodies in rats primed by prior immunization with KLH-GalGalNAc, but not in unprimed rats, indicating that the antibody response to Cj infection may depend, in part, on immune memory or the presence of pre-existing antigen-specific B-cells [97].

The IgG anti-ganglioside antibodies in GBS are of diverse origin [90] and belong to the IgG1 and IgG3 subclasses, consistent with an antigen specific T-cell driven immune response [98,99,100]. Cencioni and colleagues [101] reported that T-cells from patients with acute motor axonal neuropathy showed CD1 restricted reactivity to GM1 or GD1b gangliosides, and others reported an association between GBS and CD1 and FcɤR gene polymorphisms, which might have a role in the development of immune reactivity [102]. There are also IgM antibodies frequently present, which may be T-cell independent or precede an IgG response, but these may be insufficient to cause GBS in the absence of reactive T-cells that can disrupt the blood–nerve barrier. As an example, Rees and colleagues [39] reported that some patients with uncomplicated Cj infection without neuropathy developed IgM but not IgG anti-ganglioside antibodies.

## 10. Conclusions

Cj infection has been shown to activate multiple innate and adoptive pro-inflammatory pathways that can help overcome tolerance and induce autoimmunity [103] Elucidation of the specific mechanisms and interactions that are responsible for the development of anti-ganglioside antibodies and neuropathy following Cj infection would enhance our understanding of the relation between infection and autoimmunity and the specific mechanism responsible for neuropathy. The research would also help develop more effective preventive interventions and therapies.

## Data Availability

Not applicable.
