# Correction: Latov, N. *Campylobacter jejuni* Infection, Anti-Ganglioside Antibodies, and Neuropathy. *Microorganisms* 2022, *10*, 2139

**DOI:** 10.3390/microorganisms11030640

**Published:** 2023-03-02

**Authors:** Norman Latov

**Affiliations:** Department of Neurology, Weill Cornell Medicine, 1305 York Ave, Ste 217, New York, NY 10021, USA; nol2002@med.cornell.edu

The authors wish to make the following corrections to this paper [1]:

In the last paragraph of Section 9, Immune Mechanisms and Induction of Ant-Ganglioside Antibodies, the author mistakenly wrote: “As an example, Hirano and colleagues [84] reported on a family with Cj infection, in which IgG but IgM anti-ganglioside antibodies were associated with GBS.” 

The correct version should be as follows: 

“As an example, Rees and colleagues [39] reported that some patients with uncomplicated Cj infection without neuropathy developed IgM but not IgG anti-ganglioside antibodies.”

The authors apologize for any inconvenience caused and state that the scientific conclusions are unaffected. The original publication has also been updated.
